# Donors, authors, and owners: how is genomic citizen science addressing interests in research outputs?

**DOI:** 10.1186/s12910-019-0419-1

**Published:** 2019-11-21

**Authors:** Christi J. Guerrini, Meaganne Lewellyn, Mary A. Majumder, Meredith Trejo, Isabel Canfield, Amy L. McGuire

**Affiliations:** Baylor College of Medicine, Center for Medical Ethics and Health Policy, 1 Baylor Plaza, Houston, TX 77030 USA

**Keywords:** Citizen science, Intellectual property, Return of results, Research ethics, Direct-to-consumer genetic testing

## Abstract

**Background:**

Citizen science is increasingly prevalent in the biomedical sciences, including the field of human genomics. Genomic citizen science initiatives present new opportunities to engage individuals in scientific discovery, but they also are provoking new questions regarding who owns the outputs of the research, including intangible ideas and discoveries and tangible writings, tools, technologies, and products. The legal and ethical claims of participants to research outputs become stronger—and also more likely to conflict with those of institution-based researchers and other stakeholders—as participants become more involved, quantitatively and qualitatively, in the research process. It is not yet known, however, how genomic citizen science initiatives are managing the interests of their participants in accessing and controlling research outputs in practice. To help fill this gap, we conducted an in-depth review of relevant policies and practices of U.S.-based genomic citizen science initiatives.

**Methods:**

We queried the peer-reviewed literature and grey literature to identify 22 genomic citizen science initiatives that satisfied six inclusion criteria. A data collection form was used to capture initiative features, policies, and practices relevant to participants’ access to and control over research outputs.

**Results:**

This analysis revealed that the genomic citizen science landscape is diverse and includes many initiatives that do not have institutional affiliations. Two trends that are in apparent tension were identified: commercialization and operationalization of a philosophy of openness. While most initiatives supported participants’ access to research outputs, including datasets and published findings, none supported participants’ control over results via intellectual property, licensing, or commercialization rights. However, several initiatives disclaimed their own rights to profit from outputs.

**Conclusions:**

There are opportunities for citizen science initiatives to incorporate more features that support participants’ access to and control over research outputs, consistent with their specific objectives, operations, and technical capabilities.

## Background

Citizen science initiatives have exploded in recent decades and now number in the thousands worldwide [1]. The definition of citizen science is contested and evolving [2–5], but it generally refers to an approach to scientific inquiry in which members of the public participate in one or more steps of the research process other than, or in addition to, allowing personal data or biospecimens to be collected from them for analysis by others. Guides, tools, and templates that support citizen science initiatives from the planning stages through evaluation are now widely available, and organizations have emerged around the world to support and promote these efforts [6].

Although citizen science is deeply rooted in ecology and the environmental sciences, it is increasingly prevalent in the biomedical sciences, including the field of human genomics [7]. Gene-related citizen science initiatives are diverse and involve citizen scientists in activities that include annotating scientific resources; collecting, publishing, and analyzing genetic data; and designing and executing studies—sometimes using themselves as research subjects. That genomic citizen science is flourishing should not be surprising given growing public interest in the genetic bases of health, traits, and habits and the development of new tools and services that facilitate personal genetic discovery and data sharing [8].

At the same time that expansion of the genomic citizen science landscape is presenting new opportunities to engage individuals in research, it also is provoking complex questions regarding who owns the outputs of the research process. In the United States, statutes, regulations, and case law address the rights of participants in biomedical research to their research *inputs*—i.e., the data and biospecimens they contribute for analysis. While some states have passed laws declaring that genetic information is the property of the individual to whom it pertains [9], courts have generally been reluctant to find that individuals who provide their data and biospecimens to researchers retain property rights in those resources [10–12]. Nevertheless, alternatives to this default position can be negotiated, and in fact, a number of citizen science initiatives involving human genetic data have adopted policies or follow practices that respect the continuing interests of participants in their research inputs.

This article refers to those interests as *ownership interests*. Importantly, we do not use that term to refer only to legal property rights. Rather, we conceptualize ownership interests consistent with a metaphor of property as webs of interests involving potentially many different individuals, groups, and entities, each of whom has a relationship with some tangible or intangible thing [13]. These relationships form the basis of interests in that thing, where interests include rights, expectations, responsibilities, and powers [13]. But “[i] ntense feelings of ownership can exist in the absence of legal ownership” [14]; some of these interests, even if justified, do not qualify for legal protection [13]. Nevertheless, ownership interests in a thing might be recognized or validated outside of traditional property law in a number of ways, including by operation of policies or practices that grant individuals exclusive or non-exclusive access to or control over that thing. For example, allowing participants to determine who will have access to the data and biospecimens they contribute, and for what purposes, are practices that recognize or validate research participants’ ownership interests in their research inputs.

While scholars have described conceptual, practical, and legal challenges associated with propertization of personal health data [15–17], other problems are raised when genomic citizen science recognizes or validates participants’ ownership interests in the *outputs* of research. By research outputs, we mean all intangible ideas and discoveries, as well as tangible writings, tools, technologies, and products, that result from the research process. Some of these outputs might be protected by contract law when, for example, rights to commercialize outputs are negotiated.

Outputs might also be protected by intellectual property (IP) law, which encompasses copyrights and patents. In the United States, original works fixed in any tangible medium of expression are protected by copyright law [18]. Copyright vests automatically in the author of such a work, who enjoys broad ownership rights to exclude others from reproducing and distributing the work and creating derivative works [18]. When two or more authors merge their contributions into a single work, they are considered co-authors and have an equal, undivided interest in the work as a whole [19]. A patent, on the other hand, is a legal right to exclude others from making, using, offering to sell, or selling the invention described in the patent during the patent term [20]. The legal inventors of a patentable discovery are the individuals who contributed to its conception, and individuals may be co-inventors even if they did not physically work together or at the same time and even if they did not make the same kinds of contributions [21, 22]. Ownership of a patent initially vests in the inventors [22]. However, inventors can agree to assign, or legally transfer, their ownership rights to others [20].

Citizen science initiatives for which openness is a priority might publicly disclose all research outputs and require individuals to surrender their ownership interests in those outputs—including IP interests—as a condition of participation. Alternatively, initiatives might recognize or validate participants’ ownership interests in research outputs by, for example, inviting participants to co-author and therefore obtain copyrights in publications that report outputs; providing participants access to outputs, such as datasets or research findings; giving participants a say in the disclosure or patenting of outputs; or even promising participants a percentage of profits from licensing or commercializing outputs.

Over the years, there has emerged a general consensus that citizen science initiatives should recognize or validate participants’ ownership interests in research outputs in certain ways. For example, the European Citizen Science Association (ECSA) has articulated key principles (also described as best practices) of citizen science that include providing access to project datasets and informing participants of research outcomes [23]. These principles are consistent with citizen science’s characterization as an “example” of “open science,” which is a movement to make scientific data and outputs publicly accessible [24]. Scholars agree that, especially where participants are “co-producers” of research data, they might “have a valid stake in the ownership of that data” that at least merits informing participants about data use [25]. Meanwhile, a review of 32 published articles reporting participants’ preferences for communication of citizen science research outputs found consistent evidence that participants value access to datasets created by, and the scientific outcomes of, the research they support [26]. The articles further reported that access benefits both citizen science initiatives and participants by motivating initial, continued, and renewed participation and increasing participants’ scientific understanding of and engagement in research processes [26].

Another ECSA core principle urges leaders of citizen science initiatives to take into consideration ethical issues relevant to IP [23]. Some scholars recommend that initiatives not only clarify IP rights with their participants, but also that they consider negotiating relevant agreements to ensure that all stakeholders receive a “fair share” of the benefits of the research [27]. These might include, for example, profit-sharing agreements.

Elsewhere, we (and others) have described challenges associated with managing ownership interests in citizen science outputs [27–29]. Among other things, participants’ legal and ethical claims to research outputs become stronger—and also perhaps more likely to conflict with the interests of researchers and other stakeholders—as participants become more involved, quantitatively and qualitatively, in the research process [28, 30]. It is not yet known, however, how citizen science initiatives are managing participants’ ownership interests in practice and whether they are doing so in ways that are consistent with ethical consensus and participants’ preferences and values. Our aim was to help fill this gap by conducting an in-depth review of relevant policies and practices of U.S.-based genomic citizen science initiatives.

The study was limited to genomic initiatives for three reasons. First, as mentioned earlier, some of these initiatives already recognize or validate participants’ ownership interests in their research inputs. Therefore it seemed reasonable to conclude that they might also have given serious consideration to participants’ interests in research outputs. Second, there is legal precedent for recognizing the ownership interests of communities in research that uses their genetic data. For example, the Nagoya Protocol (NP) requires researchers to share the benefits arising from their activities with local communities from which nonhuman genetic resources and traditional knowledge associated with nonhuman genetic resources are collected [31]. Acceptable nonmonetary benefits recognized by the NP include access to research results, participation in product development, and joint ownership of relevant IP rights [31]. Although the United States is not a signatory to the NP, it reflects growing international consensus that individuals and communities with close ties to genetic resources that are used in genomic research can have compelling interests in research outputs that merit recognition or validation.

Third, there have been instances in which individuals who contributed personal genetic data for research purposes directly challenged assertions of control over research outputs. In 2012, for example, customers of the direct-to-consumer (DTC) genetic testing company 23andMe who had given the company permission to use their genetic data for research expressed dismay upon learning that it had procured a patent related to Parkinson’s disease using their data [32]. Their concern was that 23andMe would use the patent to charge royalties or block the performance of genetic testing, inconsistent with the company’s avowed mission of democratizing genomics and the customers’ belief that they were helping patients [32, 33]. Families affected by Canavan disease similarly objected to researchers’ patenting of diagnostic tests that were developed using funds and resources provided by the families [11]. When the families learned that the patents were being used to restrict access to testing, they filed a lawsuit alleging, among other things, the researchers’ unjust enrichment [11, 34].

For all of these reasons, we hypothesized that it was likely that at least some genomic citizen science initiatives have given serious consideration to how to manage their participants’ interests in research outputs. If so, information about the approaches they are using might be broadly useful to citizen science initiatives that are only just beginning to consider these issues.

## Methods

### Dataset construction

Eligible initiatives were identified by querying the peer-reviewed literature as well as the grey literature using synonyms of the terms “citizen science” and “genetic.” This search was repeated at intervals between February 2017 and July 2018. Websites that connect citizen scientists to specific projects (e.g., SciStarter and Zooniverse), educational websites (e.g., Citizen Science Center), blogs (e.g., BYOBio.net), and genomic news outlets (e.g., GenomeWeb) also were scanned throughout the data collection period. Additional initiatives were identified through professional networks and other research activities.

Eligible initiatives were defined broadly to include any project, platform, study, or service, without regard to its institutional affiliation(s) or commercial status, that satisfied six criteria at the time of evaluation. First, initiatives must have solicited the public to: contribute personal genetic information for specific or non-specific research purposes; assist in designing or executing research using genetic information derived from human biospecimens; or crowdsource analysis or management of such information. By accommodating a variety of contributions, this criterion is consistent with the general understanding that the term “citizen science” encompasses diverse study designs, including data donation models [35]. On the other hand, by limiting eligible initiatives to those involving human genetic information, application of this criterion had the effect of excluding projects focused on the genetic information of microbes, plants, and other animals, as well as online citizen science games that involved sequence information presented in the abstract.

The next criteria were directed to feasibility. Specifically, we excluded any initiative that did not have corporate headquarters or an office in the United States. Because IP practices and policies that are adopted by individuals and entities are influenced to some extent by the domestic IP laws that govern them, the dataset was restricted to U.S.-based initiatives governed by the IP laws with which we are most familiar. Third, we required sufficient information available online to understand each initiative’s objectives, functions, and mechanisms for addressing ownership interests in research outputs.

Focusing on the “science” of citizen science, the fourth criterion was that each initiative conduct or otherwise facilitate research designed to have what ECSA describes as a “genuine science outcome” [23]. This criterion was satisfied by tools and services that both generated information for individuals based on their personal data and stated that they were conducting or facilitating, or had plans to conduct or facilitate, research using those data. Tools and services that only generated individual health, trait, or wellness reports from personal genetic data were excluded. Fifth, the initiative was required to provide some benefit to participants other than money, such as personally meaningful information or scientific training. This criterion was adopted in light of the common understanding that the ethos of citizen science encompasses some degree of reciprocity [23, 25].

Finally, each initiative was required to publicly refer to itself using the rhetoric of collaboration, empowerment, or democratization that is associated with citizen science. Although some initiatives in the dataset described themselves specifically as “citizen science,” use of that term was not required given that it is not universally known and also is sometimes rejected by projects that welcome participation by noncitizens [4]. Therefore, for example, self-description as an “experimental community” or “community-owned science” satisfied this criterion. Although crowdsourcing is not exclusive to citizen science, it is used to describe a mechanism by which public participation in research is achieved [4, 36], and so self-description by reference to crowdsourcing also was sufficient. The framing of this criterion is consistent with empirical studies that identified publications describing citizen science projects based on their self-description as citizen science or using related terms [2, 37]; empirical studies that identified citizen science projects based on their decision to join citizen science platforms [38]; and case studies of participatory research groups, movements, and initiatives that were selected based on their self-description as participatory or labeling as participatory by others [33].

**Fig. 1:**
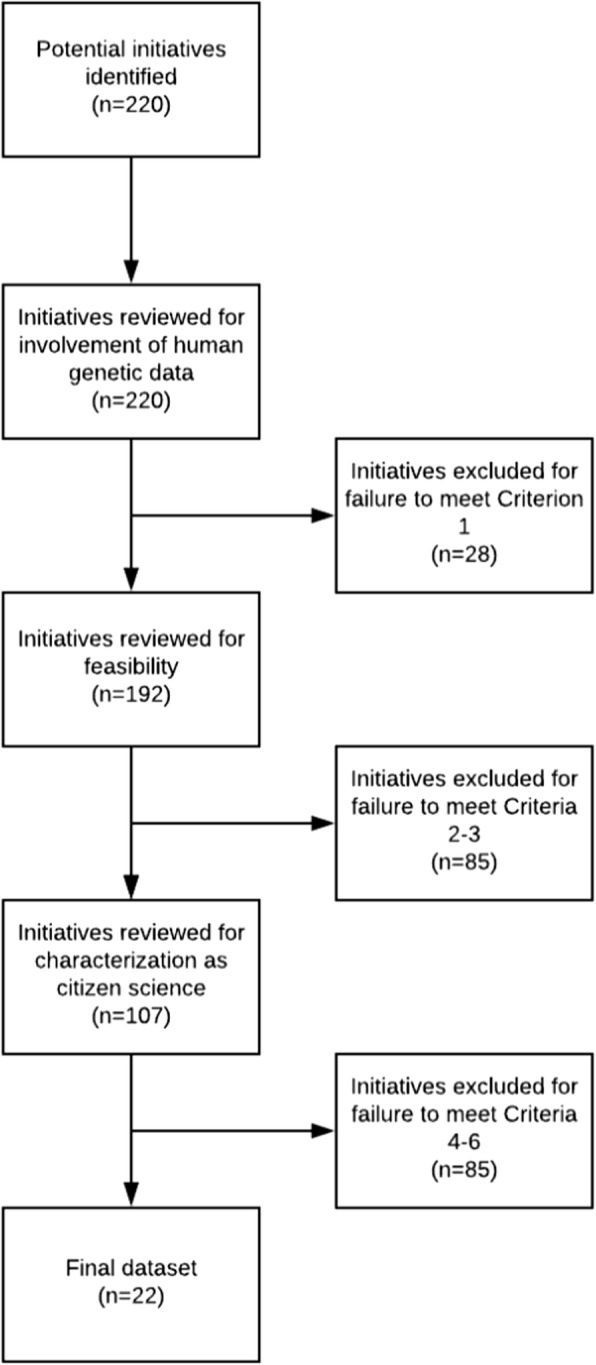
Dataset construction

Applying these criteria, we considered 220 initiatives and excluded 198 for failure to satisfy all six criteria (Fig. 1). The resulting dataset consisted of 22 initiatives (Table 1). Initiatives in four pairs were related to each other but determined to be sufficiently independent to justify separate analyses (see Additional file 1).

**Table 1:** Dataset initiatives

Genetics of Taste Lab Studies	DNAsimple	MyGene2	Genos
National Genographic Project	Genomes in Need	Open Humans	DNA.Land
Infinome	Altruist Database	openSNP	GeneKnot
Personal Genome Project	MTHFR Study	23andMe	Genevieve
DIYgenomics	Invitae Patient Insight Network	Genomes Unzipped	GET-Evidence
Spit for Science	GenomeConnect		

### Content search and analysis

For each initiative in the dataset, we searched for publicly available information regarding its organization, features, and policies and practices for managing participants’ ownership interests in research outputs. This search involved not only examining each initiative’s website and all documents published on its website, but also querying the peer-reviewed literature and grey literature for descriptions of the initiative. When potentially relevant information was identified, it was saved and stored in a cloud-based workspace. (Policies and other dynamic internet content that are quoted below refer to these archived versions.) Similar to systematic studies of third-party genetic interpretation tools [8] and DTC genetic testing and interpretation services [39], we then developed a data form to record and organize findings from analysis of the collected information. After initial development of the form, a pilot study was conducted. The form was then modified to clarify data categories.

Population of the final data collection form was conducted by two authors (CG, ML). First, information about each initiative’s age, structure, and features relevant to participant contributions was recorded. These features included the kinds of contributions that the initiative accepted or required: biospecimens for genetic testing; personal genetic information, which was broadly defined to include genetic data and genetic testing reports; survey information; social media or fitness data; and analyses. We also recorded benefits that individuals could receive as a result of participation that were not related to access to or control over research outputs. Each initiative was then categorized according to what we perceived to be its primary role from the perspective of its participants: research—conducting or facilitating one or more genetic research studies; interpretation—providing interpretation of individual genetic data; repository—serving as a public repository of genetic data or interpretive literature; registry—serving as a private registry of genetic data; matchmaking—matching participants to other participants or researchers; or platform—serving as a platform for communication and collaboration around genetic data.

Next, initiatives’ policies and practices related to their participants’ ownership interests in research outputs were analyzed. Consistent with the broad conceptualization of ownership described above, these interests were operationalized as exclusive or non-exclusive access to, or control over, research outputs. With respect to access, we recorded information regarding each initiative’s policies and practices related to providing participants access to data that the initiative had collected and processed, as well as the initiative’s findings and conclusions as reported in scientific publications and posters.

With respect to control, although informal mechanisms can be used to recognize or validate participants’ control over research outputs, our analysis was limited to mechanisms related to IP that could be confirmed by publicly available materials. For comparison, mechanisms that initiatives used to retain or exercise control over outputs also were captured. In the context of research outputs, copyright is most relevant to published reports that describe research objectives, processes, or results. Because it is often not possible to identify the author(s) of dynamic internet content, however, we focused on authorship rights relevant to published articles and research posters.

Another mechanism of giving participants control over the disclosure of research findings is by legally recognizing them as inventors of patentable things or ideas. Thus, initiative websites, journals, and media reports were searched for evidence that one or more participants might be inventors of related patents. If such evidence was identified, inventorship was confirmed by querying the U.S. Patent and Trademark Office’s (PTO) PAIR database using the alleged inventor’s name. In the absence of any evidence of a participant’s inventorship, however, the PAIR database was not queried. That is because most initiatives did not identify their participants by name, making it impossible to search the database to identify their patented inventions. The PTO’s Assignment Search database was also queried to identify whether any initiatives were assignees of related patents.

## Results

The results of this analysis are presented as follows. First, the initiatives in the dataset are characterized according to their objectives, organizational structures, and features. Next, mechanisms by which initiatives recognized or validated participants’ interests in accessing and controlling research outputs are described. For comparison, we also describe mechanisms used by initiatives relevant to their own control of research outputs.

### Initiatives

The 22 initiatives in the dataset exemplify the diversity and dynamism that are said to be characteristic of citizen science generally (Table 2). Initiatives were launched between 2005 and 2017, with eight (36%) coming online after 2014. At the time of review, at least four appeared to be inactive or have concluded (Genomes in Need, DIYgenomics, the MTHFR Study, and Genomes Unzipped), although their online profiles remained intact. Initiatives that had commercial affiliations, or no institutional affiliations at all, accounted for more than half of the dataset (*n* = 12, 55%). All but one of the commercial initiatives (23andMe) launched after 2014.

**Table 2:** Characteristics of dataset initiatives (*N* = 22)

*Characteristic*	n (%*)
Primary role	
Research	3 (14%)
Interpretation	7 (32%)
Repository	5 (23%)
Registry	2 (9%)
Matchmaking	2 (9%)
Platform	3 (14%)
Date launched	
Before 2011	8 (36%)
2011 to 2014	6 (27%)
2015 to 2018	8 (36%)
Primary institutional affiliation	
Commercial	6 (27%)
Academic	5 (23%)
Governmental	1 (5%)
Independent non-profit	4 (18%)
None	6 (27%)
Potential financial implications**	
Free to participate	16 (73%)
Cost to participate	6 (27%)
Paid to participate	4 (18%)
Potential inputs**	
Biospecimens	8 (36%)
Genetic data	22 (100%)
Survey data	14 (64%)
Social media or fitness data	5 (23%)
Analysis	10 (45%)
Potential non-IP returns**	
Raw genetic data	5 (23%)
Ancestry or relative information	5 (23%)
Health, wellness, or trait information	16 (73%)
Scientific training	2 (9%)
Educational credit	2 (9%)
Research matchmaking	11 (50%)
Participant matchmaking	13 (59%)

* Due to rounding, percentages in each category may not add to 100%

** Characteristics in this category are not mutually exclusive

Even when associated with for-profit entities, most initiatives (*n* = 16, 73%) allowed at least some form of participation at no cost. When participation was not free, costs always were associated with purchasing genetic testing services (e.g., $99.99 for genotyping by 23andMe) or otherwise complying with study protocols (e.g., several hundred dollars to purchase blood tests and supplements necessary to participate in the MTHFR Study). Some initiatives offered financial inducements to participate (e.g., $50 per study offered by DNAsimple) or recruit others (e.g., $20 per recruited person offered by 23andMe).

Although most initiatives served multiple purposes, the primary role of the majority of initiatives was either to provide interpretation (*n* = 7, 32%) or serve as a public repository (*n* = 5, 23%). Every initiative (*n* = 22, 100%) accepted or required participants to contribute personal genetic information (in some cases generated from biospecimens that they provided for testing) or facilitated the transfer of such information. These data assume that community scientists participating in the Genetics of Taste Lab (GTL) Studies at the Denver Museum of Nature & Science and student scientists engaged in Spit for Science, a campus-wide initiative of Virginia Commonwealth University, were not excluded from contributing their own genetic information (generated from biospecimens) for research. Nineteen initiatives (86%) accepted or required other kinds of health information. Notably, almost half (*n* = 10, 45%) invited or required analytical inputs, such as scientific analyses of collected data (e.g., GTL Studies), data annotations (e.g., Genevieve), proposed diagnoses (e.g., Genomes in Need), or data curation suggestions (e.g., GeneKnot).

Most initiatives (*n* = 16, 73%) returned health, wellness, or trait information to at least some participants. Other common benefits offered to participants were connecting them to researchers (*n* = 11, 50%) or other participants (*n* = 13, 59%) with shared health interests. Every initiative categorized as a public data repository offered one or both matchmaking features, while every initiative that offered genetic testing gave participants the option of downloading their uninterpreted genetic data.

### Access

Half of the initiatives in the dataset (*n* = 11, 50%) provided participants, and in some cases the public, access to project data or analyses (Table 3). In addition, Spit for Science made de-identified genetic data available to students enrolled in particular programs for the purpose of designing and executing studies using those data.

**Table 3:** Dataset initiatives’ mechanisms related to access to research outputs

*Access domains*		*Initiative(s)*	
Participant access to data or analyses		GTL Studies	Open Humans
		Personal Genome Project	openSNP
		Genomes in Need	Genomes Unzipped
		Altruist Database	Genevieve
		MTHFR Study	GET-Evidence
		MyGene2	Spit for Science (limited)
Participant access to findings and conclusions	Publications	GTL Studies	23andMe
		Personal Genome Project	Spit for Science
		MTHFR Study	GET-Evidence
		openSNP	GenomeConnect
	Posters	Spit for Science	GenomeConnect
	Other	Spit for Science (presentations)	23andMe (identification of articles reporting research that used personal data)
		Open Humans (policy encouraging open access publication)	

With respect to access to initiative findings and conclusions, we were unable to identify any journal articles for half of the initiatives in the dataset at the time of review. Eight initiatives (36%) that did engage in journal publication, however, identified at least some of those articles on project websites or blogs and usually also included direct links to them. Moreover, almost all of the identified articles were open-access and not located behind paywalls. In addition, two initiatives (9%)—GenomeConnect, a government-sponsored registry of genetic data that facilitates matches among participants, and Spit for Science—published research posters on their websites.

Initiatives supported participants’ access to research outputs in still other ways. Open Humans, for one, adopted a guideline applicable to projects hosted on its platform that “encourage[d] open access publication whenever possible.” Meanwhile, 23andMe launched a feature for customers who opted to contribute their data for research purposes that identified every publication reporting study results that used their personal data. Finally, Spit for Science disseminated findings and conclusions directly to participants and potentially interested communities at town halls and in lecture series, podcasts, and regular contributions to the Stall Seat Journal, a publication produced by the university’s wellness center and placed in restrooms across campus.

### Control

Of the dataset initiatives that engaged in publishing, three included participants as co-authors (Table 4). Leaders of the GTL Studies co-authored a published paper with a community scientist [40], and five of the seven participant-leaders in the MTHFR Study co-authored a paper reporting the results [41]. In addition, several student participants in Spit for Science co-authored articles reporting results of their scientific analyses [42–44].

**Table 4:** Dataset initiatives’ mechanisms related to control over research outputs

*Locus of control*	*Control domains*	*Initiative(s)*	
Participant control	Co-authorship	GTL Studies	Spit for Science
		MTHFR Study	
	Patent inventorship	*None*	
	Waiver of IP rights	GTL Studies	GenomeConnect (limited)
	Waiver of commercialization rights	DNAsimple	Spit for Science
		Personal Genome Project	Genos
		Altruist Database	DNA.Land
		23andMe	GenomeConnect
		Genomes Unzipped	
Initiative control	Patent ownership	23andMe	
	Disclaimer of patent rights	MyGene2	
	Disclaimer of profits from participant contributions	Personal Genome Project	Genomes Unzipped
		Altruist Database	Patient Insight Network (limited)
		MyGene2	DNAsimple (limited)
		openSNP	

Genomes Unzipped, a “public experiment” in which 12 individuals released their genetic data on a website with an objective of supporting scientific inquiry and developing tools for genetic analysis, was the only initiative that had adopted a formal policy on authorship. That policy, embedded within a participant agreement, explained that authorship of any publications would depend on individual analytical inputs and drafting contributions. However, we did not identify any peer-reviewed articles about the project authored by the participants.

We also did not identify any instance of participant inventorship. However, we found evidence suggesting that two initiatives required participants to assign their patent rights as a condition of participation. Specifically, GTL’s website indicated that its community scientists were required to sign a general volunteer agreement with the Denver Museum of Nature & Science that included their assignment of rights to any IP created on behalf of the museum. Meanwhile, the privacy policy of GenomeConnect provided that participants had no right to inventions or discoveries related to the initiative.

By comparison, only one initiative (23andMe) disclosed on its website that it owned patents. In addition, we identified two sponsors of three other initiatives (Sequencing.com and Invitae) as assignees of patents or patent applications, although this IP was not necessarily related to the outputs of either company’s citizen science initiatives. Nine initiatives (41%) had adopted policies excluding participants from sharing profits from discoveries facilitated by their participation. The research matchmaker Genos, for example, implied consent by anyone who submitted saliva samples for genetic sequencing to Terms of Service that included an explicit “waiver of property rights.” According to that waiver, participants agreed that, by providing a saliva sample, having their genetic data processed, or accessing their genetic data, they acquired no rights in and would not be compensated for research or commercial products developed from use of their genetic data. Similarly, the Participant Information Form for Genomes Unzipped, one of the first initiatives to publish participants’ individual genetic data online, required participants to acknowledge that neither they nor their heirs would “gain financially from any discoveries, whether or not of a commercial nature, made using the information and/or specimens” that they provided.

On the other hand, one initiative—MyGene2—made a public commitment not to pursue patents. In particular, its Ethics Framework stated that the initiative would not “make priority claims to data or discoveries.” This would appear to encompass patents since a patent is based on a claim of priority to discovery of an invention. In addition, seven initiatives (32%) made commitments not to attempt to monetize participants’ inputs in at least some circumstances, with some disclaiming any attempts to profit. For example, MyGene2’s Ethics Framework included a statement that it would “not try to monetize or profit from any facet of the gene discovery process.” Meanwhile, both Genomes Unzipped and the Personal Genome Project stated that they would not license participants’ data or biospecimens for financial gain or profit, although the Personal Genome Project noted that secondary researchers might try to do so. As yet another example, openSNP, a public repository of genetic data that connected users to information relevant to their genetic variants, announced its renunciation of profits as follows: “This project is not about making money, selling data or to quote Google: ‘We don’t wanna be evil.’ We are just interested in making science more open and accessible.”

## Discussion

Given the evolving nature of citizen science and the unique challenges associated with designating an initiative as “citizen science,” this analysis was not designed to be an exhaustive account of genomic citizen science initiatives. Rather, the aim was to conduct a systematic study from which to identify common approaches and trends. Although the dataset was relatively small, it nevertheless captured significant diversity in the genomic citizen science landscape. The profile of each initiative in the dataset was unique in terms of its design, objectives, and functions, which ranged from conducting traditional genetic research, to publishing genetic data for annotation and analysis, to matching individuals with shared research interests. Perhaps notably, almost half of the initiatives in the dataset (*n* = 10, 45%) were developed outside of academic, commercial, or governmental institutions. For example, Genomes Unzipped and the MTHFR Study, an intervention designed by individuals with a specific genetic variant to test the effect of different vitamin regimens on their homocysteine levels, were grassroots efforts of individuals who designed and executed the initiatives entirely in their private rather than professional capacities.

Two trends in genomic citizen science were tentatively identified. The first is increasing commercialization of genomic citizen science initiatives. Specifically, five of the six initiatives having commercial affiliations were launched after 2014 and also account for the majority of post-2014 initiatives. Three were patient outreach arms of commercial firms and two were among the new breed of research brokers that match individuals to genomic researchers based on their genetic data or conditions. Most companies emphasized participants’ ownership of their genetic data on their websites and also the privacy and security of their storage and exchange platforms. Given survey findings that the public is less likely to trust researchers affiliated with pharmaceutical and other commercial firms than academic and nonprofit researchers [45–47], it is plausible that these messages were intended to promote participant trust.

The extent to which commercial interests have crept into citizen science conducted in other disciplines, such as environmental, space, or library sciences, is unclear, but it would not be surprising if this trend was especially strong in genomic citizen science given the significant commercial investments in genomic translational research and technology development [48] and the observations of a strong entrepreneurial spirit among citizen scientists working in the life sciences [36]. Regardless, a possible trend towards commercialization in genomic citizen science supports prioritization of important questions that are potentially relevant to citizen science conducted in any field. Among them: what is the proper role of commercial interests in citizen science? It may be that acting on those interests in specific ways—such as by selling products to participants that were developed from their inputs—gives rise to new ethical obligations that initiatives may have to their participants. For example, it might be argued that in such circumstances, participants are entitled to advance, unrestricted, or free access to those products.

Alternatively, the possible trend of commercialization in genomic citizen science may reflect misappropriation of the participatory language of citizen science by companies providing personal genetic services that also conduct research. If so, as a result of application of our sixth criterion, for-profit firms may be overrepresented in our dataset. As noted by other scholars, it is likely that some research initiatives strategically describe their activities using the “populist rhetoric” of citizen science to increase public support or mobilize participation [49]. But inappropriate use of this rhetoric also might stem from confusion regarding what counts as citizen science, which is understandable given the lack of consensus on the meaning of that term [2–4]. One group of scholars reporting on “participatory research” articles related to rural livelihoods (who used citizen science as a synonym for such research) explained that misidentification was “not necessarily nefarious,” providing as an example that when researchers interacted with farmers for the first time, “they may believe they have done participatory research even though they have not according to many definitions” [50].

The emphasis on privacy and security in the commercial sector is in tension with the second trend we identified of data openness. More than half of initiatives in the dataset provided access to project data or analyses. Some initiatives—and, in particular, those having no institutional affiliations—even practiced what might be called radical openness through requirements that participants make their personal genetic data or analytical contributions publicly available, sometimes via a Creative Commons Zero (CC0) license that operates as a complete waiver of all copyright and related rights [51]. In many cases, these features appear to have been intended (at least in part) as an expression of ideological support for open science. For example, the publication of participants’ data (identified by name) was a defining feature of Genomes Unzipped, which explained that participants chose to disclose their data because as “proponents of open data access most of us believe that doing good science means releasing complete data for others to investigate.”

Initiative features that operationalize a philosophy of openness might have the effect of limiting participation to those who have fewer privacy concerns. Alternatively, participants might be aware of potential privacy intrusions but be more willing to risk them in exchange for the potential social and scientific benefits that they believe are facilitated by public data sharing [52]. Consideration of these privacy narratives in the context of genomic citizen science merits special attention given the need for diverse participation in biomedical research to increase the generalizability of results, yet also growing awareness of the potential secondary uses of personal genetic data to, among other things, discriminate against individuals or identify them or their genetic relatives as criminal suspects. Communities or groups that have been the subject of stigmatizing research, such as the Havasupai Tribe [53], or are disproportionately represented in the criminal justice system, such as African Americans [54], may be especially reluctant to participate in genomic citizen science initiatives that disclose individual-level genetic data, even if de-identified.

Other features related to openness include providing participants access to publications reporting research findings and conclusions. Publication in peer-reviewed journals is not a priority for all citizen science initiatives [55]. For example, one study of 490 citizen science initiatives found that only 78 had generated scientific publications [2]. It is therefore not surprising that we were unable to identify any journal articles for half of the initiatives in the dataset. However, almost 75% of initiatives that authored publications facilitated participant access to them. In many cases, the articles were published in open access journals, which can charge fees of thousands of dollars per article [56]. The payment terms for the articles we identified are not known; fee reductions or waivers may have been provided. It is possible, however, that even more articles would have been published, especially by noncommercial initiatives, if open access journals adopted payment policies that are more friendly to citizen science budgets.

Open access fees should pose less of a problem for commercial initiatives, some of whom are uniquely positioned to promote access in still other ways. For example, initiatives that develop private, centrally managed datasets might inform participants of the research studies that used their genomic data. This practice is now followed by 23andMe and it is intrinsic to research broker models that obtain participants’ permission for each proposed research use of their data. Especially when commercial initiatives are profiting from participants’ contributions, there may be a compelling ethical argument that they should follow such practices when possible.

There were fewer instances of policies or practices adopted by genomic citizen science initiatives that supported participants’ control over research outputs. While co-authorship by participants (identified as individuals, groups, or teams) is not unusual for citizen science initiatives designed as online games [57–59] and is specifically encouraged in collaborative research contexts involving indigenous communities [60], co-authorship does not appear to be common across all forms of genomic citizen science. In some cases, this may be a function of design features such as anonymous participation that discourage invitations to co-author. It is possible, however, that we missed some instances of co-authorship given that information confirming an author’s status as a citizen scientist is not always easy to find. Perhaps notably, of the three initiatives that we identified as engaged in co-authorship with citizen scientists, two required in-person participation. Meanwhile, participants that are not trained to write for academic audiences may be unable to satisfy journals’ authorship criteria that require participation in manuscript preparation [50, 55]. On the other hand, some citizen scientists may be declining opportunities for authorship due to lack of interest [55].

But such opportunities never materialize when initiatives are themselves uninterested in traditional publication. Moreover, authorship opportunities are not realized when articles are rejected due to inability to satisfy requirements that are incompatible with some forms of citizen science. One such requirement is that the research be approved by an institutional review board (IRB). The Federal Policy for the Protection of Human Subjects (Common Rule) [61] and U.S. Food and Drug Administration regulations [62] require IRBs to evaluate research protocols according to standards derived from the ethical principles of respect for persons, beneficence, and justice. These regulations generally do not apply to citizen science initiatives that are conducted outside of traditional scientific institutions and do not constitute regulated clinical trials [28]. Nevertheless, many peer-reviewed journals will not publish results of research involving human participants that have not been IRB-approved [63, 64]. Initiatives that do not have access to a research institution’s IRB and cannot afford the fees charged by independent, for-profit boards [64] may not be able to place their articles with traditional journals.

In general, the dataset initiatives did not support participants’ claims to patent rights and profits. Although we are aware of other contexts in which citizen scientists are named inventors on patents [65], none of the initiatives in our dataset named participants as patent inventors, although overall patent activity was low. Moreover, none had adopted policies that recognized participants’ interests in discoveries that they might help conceive, including profits from licensing or commercializing those discoveries. To the contrary, almost half of initiatives required participants to disclaim any rights to IP or profits as a condition of participation. It is possible, however, that some of these policies might not have been followed in practice.

In apparent solidarity, some initiatives that required participants to waive IP or profits also publicly waived their own rights to IP and (at least some) profits. Mutual waivers might be considered an ethically appropriate alternative to recognizing IP or profit interests of participants: either the initiative and participants enjoy the same rights and benefits, or none of them enjoys those rights and benefits. However, some initiatives that adopted a mutual-waiver model noted that third parties might themselves attempt to patent or profit from research outputs, reflecting the reality that the potential universe of end users of genomic citizen science outputs is broad and may include some with commercial interests, even if the initiative itself has none.

### Limitations

This study’s findings and conclusions are subject to several limitations. First, the genomic citizen science landscape is dynamic. There is a steady stream of initiatives entering and leaving the landscape. For example, after the study closed, several genomic research brokers that use citizen science rhetoric launched services, and one of them—LunaDNA—adopted a profit-sharing arrangement according to which participants receive shares in the company in exchange for their genetic data [66]. In addition, features, practices, and policies of citizen science initiatives are usually not static but evolve over time. For example, during the study period, one initiative that initially had satisfied all inclusion criteria changed from a citizen science research initiative to a commercial service for professional researchers and DTC testing companies and so ultimately was excluded from the dataset. The results reflect the activities of the landscape during a specific period of time, but because the data collection period spanned eighteen months, emerging initiatives and major changes to initiatives were identified.

Second, the activities that were captured describe only a segment of the genomic citizen science landscape. Application of study criteria resulted in the exclusion of online games because they failed the first criteria; “N of 1” studies and other activities conducted by citizen scientists working in isolation because they failed the third or fourth criteria; and studies involving patient advocacy organizations as partners in the research process because they failed the third, fifth, or sixth criteria. However, we suspect that these initiatives were unlikely to address issues of access and control using mechanisms other than those used by the initiatives in the dataset. One known exception is research supported by patient advocacy organizations, which might involve the negotiation of patent, licensing, and commercialization rights before the start of any research activities [65]. While the research inputs of some disease advocacy organizations have been empirically investigated and reported [67], the contracts they are entering to manage research outputs merit further study. However, different methodological approaches, such as qualitative interviews or surveys, may be required to obtain relevant information given that such contracts are usually not public.

Finally, our criteria resulted in the inclusion of initiatives that, according to some, do not qualify as citizen science. The participatory claims made by 23andMe in particular are often discounted [36], although one study found that the company performed well on several participatory dimensions of citizen science [33]. Ultimately, we sought to minimize subjectivity and the influence of our own biases in categorizing initiatives as citizen science by relying in good faith on the language they used to describe their ethos, objectives, and activities. However, we recognize the risk that this approach may have had the effect of validating misappropriations of citizen science rhetoric. The practical and philosophical challenges associated with identifying inclusion criteria for citizen science landscape studies should not be underestimated, and we support continued dialogue on this issue.

## Recommendations and conclusion

This analysis revealed that the genomic citizen science landscape is diverse, consistent with the significant heterogeneity in study design, objectives, and functions observed in citizen science generally. However, genomic citizen science could be unusual in the prevalence of initiatives that are sponsored by or affiliated with commercial entities or, on the other hand, not affiliated with any institutions at all. While most initiatives supported participants’ access to research outputs, including datasets and published findings, none supported participants’ control over results via IP, licensing, or commercialization rights. However, several initiatives disclaimed their own rights to patent or profit from outputs.

Although many mechanisms for recognizing or validating participants’ ownership interests in the outputs of the research they support will not be feasible or appropriate in specific contexts, genomic citizen science initiatives might have an ethical obligation to identify and implement mechanisms that are compatible with their particular objectives, capabilities, and other obligations. That is because an initiative’s self-identification as citizen science could give rise to participants’ expectations that they will benefit from the research process in ways that include access to and control over research outputs.

If so, and consistent with ethical consensus and empirical findings that participants in citizen science value knowing the scientific outcomes of the research they support [23, 25, 26], design of genomic citizen science initiatives should consider including features that support citizen science participants’ access to published results. At the same time, peer-reviewed journals might be encouraged to evaluate their requirements and fees to identify adjustments they can make that will support open-access citizen science scholarship without diminishing standards for ethical and high-quality research. Potential adjustments include providing fee waivers for citizen science projects and recognizing alternatives to IRB review, such as crowdsourced ethics review [68] or systematic ethical reflection by participants [69]. Genomic citizen science initiatives should also consider providing their participants access to project data where doing so is consistent with study design, participants’ privacy expectations, and funding and legal requirements.

In general, features relevant to participants’ control over research outputs are probably less practical to adopt given the significant problems that can arise when individuals with divergent interests are given rights of control. Although there is ethical support for incorporating such features [27], it is not yet understood which features are valued by participants or what is their effect on participation and other aspects of the research process. Studies of analogous circumstances, for example, found that only a minority of survey respondents believed that academic hospitals that sell excess biospecimens should share the profits with the public [70] and only one of 25 participants in deliberative democracy exercises felt that biobank donors should be given preferential access to treatments developed from research that used their samples [71]. These questions merit future investigation in citizen science contexts, but in the meantime, at a minimum, initiatives should consider supporting co-authorship with participants when publication is a desired research output, participants express interest in co-authorship, and resources are available to support these collaborations. Finally, initiatives might be encouraged to experiment with nonlegal mechanisms of recognizing participants’ interests in research outputs, such as providing participants a say in the disclosure and use of those outputs.

## Supplementary information

**Additional file 1.** Related genomic citizen science initiatives. This file provides additional detail regarding identification of initiatives for inclusion in the dataset.

## Data Availability

Data generated or analyzed during this study are included in this published article or are otherwise available from the corresponding author upon reasonable request.

## Abbreviations

DTC: Direct-to-consumer
GTL: Genetics of Taste Lab
IP: Intellectual property
IRB: Institutional review board
