# Supplementary material for: Donors, authors, and owners: how is genomic citizen science addressing interests in research outputs?
Source: BMC Med Ethics. 2019 Nov 21;20:84. doi: 10.1186/s12910-019-0419-1 (PMC6868686; doi:10.1186/s12910-019-0419-1)
Supplement: Supplementary file 1 — Additional file 1. Related genomic citizen science initiatives. This file provides additional detail regarding identification of initiatives for inclusion in the dataset. [file 12910_2019_419_MOESM1_ESM.docx]

**Supplemental File**

The resulting dataset consisted of 22 genomic citizen science initiatives. Initiatives in four pairs were related to each other but determined to be sufficiently independent to justify separate analyses (Suppl. Table 1).

| Supplemental Table 1. Related genomic citizen science initiatives. | |
| --- | --- |
| Initiatives | Relationship |
| Personal Genome Project (PGP)/ GET-Evidence | The PGP is a public repository of individual genetic data, medical records, and survey data. GET-Evidence is a public, crowdsourced literature repository that is used to interpret genetic data generated by PGP but also can be used to interpret data generated from other sources. |
| DIYgenomics/  MTHFR Study | DIYgenomics is a platform for collaboration on genomic studies designed and executed entirely by participations. The MTHFR Study is a research study that was facilitated on the DIYgenomics platform. |
| Genomes in Need/ Altruist Database | Genomes in Need is a platform for crowdsourcing diagnoses for specific individuals based on their genetic data. The Altruist Database is a public repository of individual data. Both are hosted by Sequencing.com. |
| Open Humans/ Genevieve | Open Humans is a platform that hosts independent research projects and allows users to contribute their genetic data, which is stored on the platform, to projects of their choice. Genevieve is a public, crowdsourced literature repository that is hosted on the Open Humans platform. |
